# Sensitization to cat allergen and its association with respiratory allergies: cross-sectional study

**DOI:** 10.1590/1516-3180.2017.0072170617

**Published:** 2017-11-06

**Authors:** Clóvis Eduardo Santos Galvão, Gustavo Silveira Graudenz, Jorge Kalil, Fábio Fernandes Morato Castro

**Affiliations:** I MD, PhD. Attending Physician, Clinical Immunology and Allergy Service, Hospital das Clínicas, Universidade de São Paulo (HCFMUSP), São Paulo (SP), Brazil.; II MD, PhD. Allergogist, Clinical Immunology and Allergy Service, Hospital das Clínicas, Universidade de São Paulo, São Paulo (SP), Brazil.; III MD. Full Professor, Clinical Immunology and Allergy Service, Hospital das Clínicas, Universidade de São Paulo, São Paulo (SP), Brazil.; IV MD, PhD. Attending Physician and Clinical Supervisor, Clinical Immunology and Allergy Service, Hospital das Clínicas, Universidade de São Paulo, São Paulo (SP), Brazil.

**Keywords:** Immunization, Fel d 1 protein, Felis domesticus [supplementary concept], Skin tests, Allergy and immunology

## Abstract

Cats are a significant source of allergens that contribute towards worsening of allergic diseases. The aim of this study was to investigate the association between sensitization to cat allergens and allergic respiratory diseases. This was an observational retrospective study based on the skin prick tests results of patients at a tertiary-level hospital in São Paulo. A total of 1,985 test results were assessed. The prevalence of sensitization to cat allergen was 20% (399 patients). Our data indicated that in this population of atopic patients, a positive skin prick test result for cat allergen was not associated significantly with a diagnosis of respiratory allergy.

## INTRODUCTION

The increasing prevalence of sensitization to household allergens like dust mites, pet dander and cockroaches is the result of changes to indoor environments induced by human action. Modern lifestyles and increased time spent in enclosed environments leads to reduction in natural ventilation and, consequently, increased concentration of and exposure to allergens.^1^^,^^2^ There is evidence showing a relationship between allergic sensitization in skin prick tests and the severity of asthma, and also showing that sensitization to allergens may precede manifestation of allergic diseases.^3^ According to some studies, cats are a significant source of allergens that contribute towards worsening of symptoms of allergic diseases and acute asthma attacks.^3^ There is great concern among the families of patients with respiratory allergies about keeping pets at home, and this concern is exacerbated when the desired pet is a cat. To our knowledge, there is no study on the Brazilian population assessing the association between cat sensitization and respiratory allergies.

The aim of this study was to investigate the association between sensitization to cat allergen and allergic respiratory diseases in subjects with respiratory allergy seen at a tertiary hospital in São Paulo, Brazil.

## METHODS

This was an observational retrospective study based on a review of the medical records of patients who underwent skin tests over a 12-month period at the allergy outpatient clinic of a tertiary-level hospital in the city of São Paulo (SP), in order to investigate respiratory allergies. Clinical and demographic data were collected from the study population, and only the patients with confirmed diagnoses of respiratory allergies (asthma, rhinitis, asthma associated with rhinitis, conjunctivitis and rhinoconjunctivitis) were considered for this study.

The diagnosis was confirmed based on a compatible clinical history and sensitization to aeroallergens together with clinical manifestations. Allergy skin tests were performed by means of the prick method, using standardized *Fel d 1* extract for cat allergen (FDA Allergenic, Brazil). Histamine and glycerol were used as positive and negative controls, respectively. Test results were considered positive (i.e. the reaction was positive) when the two largest perpendicular diameters of the wheals had an average value greater than or equal to 3 mm.

For each of the diagnoses considered, we analyzed the individuals sensitized and not sensitized to cat allergen based on the results from the prick test, in order to verify whether any of the groups analyzed had a greater proportion of patients sensitized to cats. Statistical analyses using cumulative percentages from tests for asthma, rhinitis, asthma associated with rhinitis, conjunctivitis and rhinoconjunctivitis were performed. Subsequently, we used the chi-square test to determine the P-value. Values under 0.05 were considered significant. The study protocol was approved by the local ethics committee. This study was developed as part of a larger protocol approved by our institution’s ethics committee (CAPPesp) on July 13, 2011, under research protocol number 0360/11.

## RESULTS

A total of 1,985 test results were assessed, predominantly from female patients. The characteristics of the patients according to their gender and age are shown in Table 1, along with the distribution according to sensitization to cat allergen. The general prevalence of sensitization to cat allergen was 20% (399 patients), of whom 1% were only sensitized to cats. Cat sensitization was detected in 20.4% of the asthmatic individuals; 19.5% of the rhinitis patients; 22.2% of the patients with asthma and rhinitis; 25% of the patients with conjunctivitis; and 24% of those with rhinoconjunctivitis. The proportion of sensitization to cat allergen (positive skin test) was not significantly higher among subjects with distinct diagnoses of respiratory allergy, as shown in Table 2.

**Table 1.**
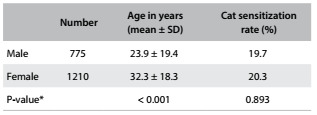
Distribution of the study population according to age and sex, and cat sensitization rate

SD = standard deviation; *Pearson’s chi square test.

**Table 2.**
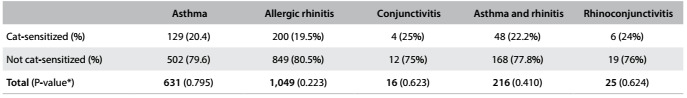
Comparison between individuals sensitized and not sensitized to cat, according to diagnoses of allergic disease

*Pearson’s chi square test.

## DISCUSSION

A total of 1,985 skin prick tests were assessed, of which 20% were positive to cat allergen. This finding is in agreement with results from studies conducted in other countries, where sensitization rates ranged from 15 to 60% in atopic patients.^1^^,^^4^ After comparative analysis between individuals sensitized and not sensitized to cat allergen, there was a higher proportion of allergic respiratory diseases in the group with positive skin tests. This result is also consistent with those of other authors.^4^^,^^5^ Gulbahar et al.^4^ investigated the prevalence of sensitization to cat dander in atopic patients and found that 44.7% had positive allergic skin test results. As in our study, sensitized patients did not show any increased risk of developing asthma. These authors observed that the prevalences of asthma in the sensitized and non-sensitized groups were 38.7% and 33.6% respectively. In our study, we found that the prevalences of asthma were 20.5% in sensitized and 31.6% in non-sensitized individuals.

The relationship between exposure to household allergens and development of sensitization and asthma has been widely studied.^2^ Although there is a general perception that sensitization to cat allergens is associated with higher prevalence of respiratory allergy, the data from the medical literature are contradictory, such that some authors have shown that the risk of sensitization, and also the prevalence of cat allergy could be increased or decreased according to the individuals’ ages and their length and level of exposure to *Fel d 1*.^5^ It is believed that the development of sensitization is influenced by several factors, including the length of exposure and the concentration of the allergen.^3^ There is no consensus in the literature regarding *Fel d 1*, or whether early exposure to it is related to high^3^^,^^4^^,^^5^ or low^2^ development of sensitization.

Studies on the concentrations of allergens that have the capacity to cause sensitization also present conflicting results. While some have suggested that there is a linear dose-response relationship, others have taken the view that low levels of *Fel d* 1 are capable of causing sensitization and that exposure to high levels can induce tolerance.^2^ Moreover, individual variability of sensitization and family histories of atopy have been reported to explain the variability of sensitization to cat allergen.

## CONCLUSION

Our data indicate that in a population of atopic patients, sensitization to cat allergen, as represented by a positive result from a skin prick test with cat allergen, was not associated significantly with a diagnosis of respiratory allergy.

## References

[1] Liccardi G, Cazzola M, D’Amato M, D’Amato G (2000). Pets and cockroaches: two increasing causes of respiratory allergy in indoor environments. Characteristics of airways sensitization and prevention strategies. Respir Med.

[2] Orysczyn MP, Annesi-Maesano I, Charpin D, Kauffmann F (2003). Allergy markers in adults in relation to the timing of pet exposure: the EGEA study. Allergy.

[3] De Vera MJ, Drapkin S, Moy JN (2003). Association of recurrent wheezing with sensitivity to cockroach allergen in inner-city children. Ann Allergy Asthma Immunol.

[4] Gulbahar O, Sin A, Mete N (2003). Sensitization to cat allergens in non-cat owner patients with respiratory allergy. Ann Allergy Asthma Immunol.

[5] Almqvist C, Egmar AC, Hedlin G (2003). Direct and indirect exposure to pets - risk of sensitization and asthma at 4 years in a birth cohort. Clin Exp Allergy.

